# Pus in the Pelvis1Read at the Buffalo Academy of Medicine, October 24, 1899.

**Published:** 1899-12

**Authors:** Herman E. Hayd

**Affiliations:** Buffalo, N. Y.


// BUFFALO MEDICAL JOURNAL.

VOL. XXXIX.—LV. DECEMBER, 1899. NO. 5.

### Original Communications.

### PUS IN THE PELVIS.[1]

BY HERMAN E. HAYD, M. D., M. R. C. S., Eng., Buffalo, N. Y.

THE pathology of Goupil and Bernutz has revolutionised the treatment of the pelvic organs, and it has taught us clinically the relationship which exists between infections of the endometrium and pus in the pelvis. Usually these inflammatory processes travel by continuity of tissue, from the endometrium to the tubes and ovaries and peritoneum, but occasionally the channel of infection is by the lymphatics of the cervix and uterus, along the broad ligament to the peritoneum, and producing the so-called true pelvic abscess, leaving the tubes and ovaries free and healthy. Consequently, we may have pus in the pelvis, as the result of an infection, perhaps gonorrheal or post abortum or post puerperal, or as a result of traumas and injuries of the cervix and body of the uterus, resulting from accidents due to careless surgical interferences or intrauterine manipulations, which may extend either along the mucous membrane to the peritoneal cavity, or by way of the lymphatics; the one producing primarily a tubal or ovarian abscess, and the other an abscess in the cellular tissue of the broad ligament.

Pus in the pelvis should be dealt with upon general surgical principles, and, like pus in any other part of the body, should be evacuated as soon as it can be with certainty diagnosticated. The history of the case, the existence of a gonorrheal infection, or a mixed infection consequent upon criminal or traumatic abortion, the presence of fever and acute pain and tenderness upon vaginal and upon

1. Read at the Buffalo Academy of Medicine, October 24, 1899.

bimanual examination, the presence of a tender mass in the pelvis, which has perhaps existed for weeks, increased leucocytosis, after having excluded pus in other quarters of the body, is strongly presumptive of a pelvic suppuration. Fever, however, does not always exist, and, in fact, is often absent, even with large collections of pus, but when present suggests an acute element in the way of an exacerbation of old inflammatory exudates, or a recent mixed infection with the streptococcus organism in evidence. Of course, the more acute and general the constitutional disturbance, the more imperative is early and active interference necessary, yet one must not lose sight of the fact that all acute inflammations of the uterus, tubes and ovaries have fever and active general disturbances, and may not at any time develop pus, but go on to perfect resolution. Nevertheless, the contrary is also true, for early in the illness pus is often formed, and, therefore, ignorance in the phenomena of these inflammations should not unnecessarily put off operative interference and leave these cases so that delay becomes responsible for a greatly increased mortality, by rendering what would be simple operative procedures absolutely desperate life-saving undertakings, with resulting post operative sequellæ and complications, often as difficult to manage as the original gross lesion.

Post puerperal infections and particularly those coming on after the second week, as a result of septic endometritis and often having latent gonorrhea present, are the cases which terminate most frequently in pyosalpinx and ovarian abscess. Dermoid cysts are often brought into pernicious activity by the puerperium and unrecognised intraligamentary and ovarian cysts frequently undergo suppuration as a result of these septic foci. Therefore, with these facts in view, an early suspicion of pus will suggest itself and the surgeon will be called to confirm these impressions wherein an early and practically safe operation will result with a speedy convalescence and a future healthy and useful life.

During the last few years, and particularly since the visit to this country of some celebrated French surgeons, the profession has been divided as to which is the best route to attack pus in the pelvis, whether by an abdominal section or through the vagina. Many of our representative operators have acted upon the suggestions of these continental teachers, and are removing by vaginal incision and drainage collections of pus, and frequently supplement this work by a vaginal hysterectomy, ablating at the same time enlarged tubes and ovaries, whether filled with pus or not. There is much to commend to us, in certain selected cases, this method of incision, but, unfortunately, a few wild enthusiasts, who seek fame by notoriety and medical journal and state society publicity, have uttered the dictum that all pus collections, whether free in the pelvis or circumscribed in the tubes or ovaries, must be attacked per vaginam. The absurdity of this sweeping position is evident to any one who has operated much from above, because he realises that it is absolutely impossible to meet with safety the mass of complications, of adhesions of bowel and omentum to pelvic viscera, as one so frequently sees in some of the desperate cases that are operated upon every day by many of us. I am free to admit that the brilliant results of our foreign surgeons have encouraged us to less radical measures sometimes, and many of us, who heretofore always operated per abdominem, now in selected cases attack per vaginam. Therefore, this question of route has much for our serious study and consideration, whether viewed from the standpoint of the vaginal hysterectomist or the abdominal sectionist, and no man can consider that he is following the course of scientific surgery, when he allies himself with any one method of operative attack to the exclusion of the other; but as his skill and diagnostic ability increases, so will he the more readily choose the proper route to best meet the diseased conditions.

Nature, by her beautiful protective processes, shields herself from the approach of pus by making dense barriers of adhesions, and, as a rule, confines the suppuration to certain definite areas. If it were possible to establish free drainage from below for all the different points of suppuration, it would be natural to expect that in a short time the acute trouble would subside and reparative processes begin; but, unfortunately, many of these pus cavities are lined by a secreting membrane and will continue to give off pus so long as these membranes exist *in situ*. Therefore, in many of these cases, the cure cannot be effected unless the diseased organ is eradicated, and if it is intensely bound down, irreparable injury must follow when operating through the narrow vagina and without the sense of sight to aid in peeling off the agglutinated organs. Again, operating through the vagina presupposes a diagnostic accuracy which men do not and never will possess, and when the advocates of this method divide their cases into certain classes, the one operable by one method, and the other by the other method, they make fast rules which practical every-day experience does not justify them in assuming.

For example, Pryor says in his recent article: "I would always consider that the vaginal is the operation of election in all cases of pelvic suppuration where the vermiform appendix did not require removal, and when there was no fistulous opening between a coil of small gut and a pus sac." Notwithstanding all that has been done by palpation in diagnosticating inflammatory conditions of the appendix, I maintain that it is not possible to say, in a given case of pelvic suppuration: in this one I shall find an appendix adherent to the pelvic mass and diseased and requiring removal, while, in the other, such a complication need not be expected. I have, times without number, delivered a large pus tube or ovarian abscess, and found that I had torn off the appendix vermiformis, or that I needed to remove it, because it was so disorganised; and this experience was not always confined to masses in the right pelvis, because occasionally I found the appendix so long, that it was densely adherent even to the left tubal and ovarian structures. So with fistulous tracts and although they most frequently exist between the pus tube and the sigmoid flexure and large bowel, yet it is impossible to make such a diagnosis before operation, and if one into the small bowels were undetected, death would surely follow, if not carefully and properly repaired. And although Pryor shows us a wonderful collection of cases—where he operated 100 times through the vagina without mortality—yet he has not convinced me that many of these women will not return for future operation, because his work through the vagina was necessarily incomplete. Price, Edebohls, Mann and a host of us lesser lights, often have a long series of cases without a death, and then, when least expected, meet a fatal issue; and I am satisfied that this element of uncertainty must be greater when we operate without the advantages of sight, which has been so much strengthened by reason of the Trendelenburg position, because it matters not how keen our sense of touch may be, by reason of repeated experiences, the educated eye and the educated fingers must together accomplish more than either could do singly.

Some cases are peculiarly suited for the vaginal operation, and all men pretty well agree that they should only be attacked through the vagina; for instance, a pelvic abscess confined to the broad ligament, or an abscess where the pus is free in the pelvis, such as I had a few days ago in the Deaconess's Hospital, or a large pyosalpinx or ovarian abscess alone, or with multiple abscesses, which has assumed such enormous proportions that it might well be called a pelvic abscess, when it exists, not only above the pelvic brim, but is even pointing low into the vagina, perhaps to the introitus. Such cases should, as I have said, be opened through the vagina and thoroughly drained, and if in the course of time, a cure is not effected, a section can be done later, when the patient's condition would justify such a procedure; or such abscesses can be thoroughly inspected by making an abdominal incision and then with the one hand in the abdomen as a guide, more effectual vaginal incisions can be made into the various abscess cavities, and all of them be more thoroughly drained. However, such a course is not often undertaken and is usually resorted to only when the abdomen has been opened, and the surgeon has thought it best to retire, because the mass was so large and so densely agglutinated to adjacent structures and the adhesions and vascular connections were so great as to make it impracticable to attempt its removal.

The advocates of vaginal operations maintain that there is less shock after such operations than when the same are performed through the abdomen. This may or may not be the case, but, so far as I am concerned, I am inclined to believe with Baldy and his followers, that shock, as we ordinarily see it, is not much of a factor in producing mortality, and that when we can exclude injuries to important organs by reason of careless manipulations, when bowel and visceral tears have been properly taken care of, when unsuspected torn ureters have been properly anastomosed, mere time consumed by experienced operators in their operations is not responsible for increased mortality. Add to this the application of methods, as Morris says, in themselves dangerous and hazardous, to meet existing complications, such as the unnecessary insertion of drainage- tubes and badly placed yards of iodoform gauze and other equally dangerous chemical products, within the peritoneal cavity, and then the mere element of time occupied in operations is still further robbed of its death possibilities.

Another argument is that hernia so frequently follows abdominal operations, which I think can be fairly met by the statement that it occurs less frequently now by reason of our improved surgical technique, and perhaps not more often than the same takes place after vaginal operations, according to Eastman, of Indianapolis, and other surgeons who have done a great deal of vaginal work.

The presence of an abdominal scar cuts no figure in this argument, unless we can prove that better, safer and more beneficial operations result from attacking diseased conditions through the vagina than above the pelvic brim, because few women care anything about their scars if good health and function have been restored to them by reason of their operation.

It is also said that the period of confinement is much less and the convalescence much more rapid when operations are performed through the vagina. This statement also interests me but little, because I am satisfied that our patients get up altogether too soon after any operation, and assume to work in an absurdly short time. I have had patients who were anxious to go home long before the end of the second week, and it was only by the strongest persuasion on my part that they could be kept in bed the prescribed fourteen days. The personal equation enters so largely into these cases, and what might with reasonable safety be permitted in one at the end of the tenth day, could not with the other, and, in all probability, both patients would best subserve nature if they rested a very much longer period. Yet Emil Ries, of Chicago, has been lately advocating the early assumption of the erect posture and the early administration of solid food after abdominal sections—beefsteak, potatoes, and the like,—in the hope of stimulating better peristalsis, and Edebohls, of New York, is advocating the early assumption of regular business duties in an absurdly short time after his appendicitis operations.

I shall not occupy your time by further dilating upon the respective advantages and disadvantages of the two methods of operating, as this was not the theme of my paper. It was pus in the pelvis, which should be let out as soon as possible; and if, by reason of your special skill and experience, you are enabled to best meet this condition through the vagina, with less danger to your patient and more satisfaction to yourself, adopt that method. But first carefully consider that vaginal operations in the pelvic cavity are more difficult to perform than through an abdominal opening, that vaginal surgery is necessarily less complete and the dangers and risks of accident to important structures infinitely greater, and the possibilities and necessities for conservative surgery of the tubes and ovaries is entirely out of the question. Whether you leave the uterus in cases of double pyosalpinx or ovarian abscess, is a subject which is not yet definitely settled, but of one thing I am sure, that the concensus of the best surgical thought of today is to leave the uterus when possible to support the lower abdominal segment, and to remove only such portion or portions of the ovaries and tubes as are irreparably diseased, so as to continue as long as possible the function of menstruation, which is necessary for the hysical and mental well being of woman.

493 Delaware Avenue.

